# Realtime Localization and Estimation of Loads on Aircraft Wings from Depth Images

**DOI:** 10.3390/s20123405

**Published:** 2020-06-16

**Authors:** Diyar Khalis Bilal, Mustafa Unel, Mehmet Yildiz, Bahattin Koc

**Affiliations:** 1 Faculty of Engineering and Natural Sciences, Sabanci University, Istanbul 34956, Turkey; diyarbilal@sabanciuniv.edu (D.K.B.); meyildiz@sabanciuniv.edu (M.Y.); bahattinkoc@sabanciuniv.edu (B.K.); 2 Integrated Manufacturing Technologies Research and Application Center, Sabanci University, Istanbul 34906, Turkey

**Keywords:** structural health monitoring, load localization, load estimation, depth sensor, artificial neural networks, castigliano’s theorem

## Abstract

This paper deals with the development of a realtime structural health monitoring system for airframe structures to localize and estimate the magnitude of the loads causing deflections to the critical components, such as wings. To this end, a framework that is based on artificial neural networks is developed where features that are extracted from a depth camera are utilized. The localization of the load is treated as a multinomial logistic classification problem and the load magnitude estimation as a logistic regression problem. The neural networks trained for classification and regression are preceded with an autoencoder, through which maximum informative data at a much smaller scale are extracted from the depth features. The effectiveness of the proposed method is validated by an experimental study performed on a composite unmanned aerial vehicle (UAV) wing subject to concentrated and distributed loads, and the results obtained by the proposed method are superior when compared with a method based on Castigliano’s theorem.

## 1. Introduction

Structural Health Monitoring (SHM) has been an increasingly important technology in monitoring the structural integrity of composite materials used in the aerospace industry. Because airframes operate under continuous external loads, they will be exposed to large deflections that may adversely affect their structural integrity. Critical components, such as fuselage and wings, should be monitored to ensure long service life. Although these components are designed to withstand different types of loading conditions, such as bending, torsion, tension, and compression, among others, a robust SHM system will be extremely valuable for the aerospace industry for realtime monitoring of loads.

Current aircraft maintenance and repair systems used for structural monitoring rely on load monitoring systems while using strain gauges [1,2], optical measurement systems [3,4,5,6,7], and fiber brag grating (FBG) [8,9,10,11] sensors.

The strain gauge based measurements are widely used both in literature and the industry for aircraft wing deflection measurements due to their ability to fit into almost any space and proven high accuracy measurements [1,2]. However, strain gauges have many limitations, such that they cannot be attached to every kind of material, they are easily affected by external temperature variations, and physical scratches or cuts can easily damage them. More importantly, a large number of them need to be installed if one needs to monitor the whole wing due to their small size.

Besides strain gauges, various approaches that were based on optical methods were investigated in the literature for measurement of wing deflections and loads acting on them. Burner et al. [3] presented the theoretical foundations of video grammetric model deflection (VMD) measurement technique, which was implemented by National Aeronautics and Space Administration (NASA) for wind tunnel testing [4]. Afterwards, many research on wing deflection measurement and analysis were motivated by the catastrophic failure of the unmanned aerial vehicle (UAV) Helios [5,6]. Lizotte et al. [7] proposed estimation of aircraft structural loads based on wing deflection measurements. Their approach is based on the installation of infrared lightemitting diodes (LEDs) on the wings; however, the deflection measurements are local, which do not cover the whole wing structure unless a large number of LEDs are installed.

Motivated by the catastrophic failure of Helios, a realtime in flight wing deflection monitoring of Ikhana and Global Observer UAVs were performed by Richards et al. [8] by utilizing the spatial resolution and equal spacing of FBGs. Moreover, FBGs have also been used by Alvarenga et al. [9] for realtime wing deflection measurements on lightweight UAVs. Additionally, chord wise strain distribution measurements that were obtained from a network of FBG sensors were also used by Ciminello et al. [10] for development of an in flight shape monitoring system as a part of the European Smart Intelligent Aircraft project. More recently, Nicolas et al. [11] proposed the usage of FBG sensors for determination of wing deflection shape as well as the associated out of plane load magnitudes causing such deflections. To simulate in flight loading conditions, both concentrated and distributed loads were applied on the wings each with incrementally increasing loads. They reported that their calculated out of plane displacements and load magnitudes were within 4.2% of the actual measured data by strain gauges. As seen from these works in literature, even though FBGs used for SHM purposes have advantages over conventional sensors, they are still highly affected by temperature changes. Moreover, their installation is not an easy task due to their fragile nature, and special attention must be given to the problems of ingress and egress of the optical fibers [12].

Although the aforementioned sensors used in the literature can be used for load monitoring in aerospace vehicles, better technologies are needed to achieve usable sensitivity, robustness, and high resolution requirements. The need for the use of a large quantity of sensors to cover the whole structure is one of the major drawbacks of these sensor technologies. Therefore, a sensor that is capable of full field load measurement from a single unit with high accuracy and precision can become an important alternative. This will also result in a considerable reduction in costs, especially when a fleet of airframes need to be inspected and monitored.

From these works in the literature, it is observed that, in general, a mathematical model for describing the deflections of an aircraft wing is used to study its behavior under different types of loads. However, obtaining physics based models of systems can easily become a difficult problem due to system complexity and uncertainties; thus, effectively decreasing their usefulness. This is especially the case in systems, where lots of data are obtained using different types of sensors, which, in turn, adds more complexity to the system due to inherent sensor noise. In such cases data driven modeling techniques have been found to be more effective since the acquired data already contains all kinds of uncertainties, sensor errors and sensor noise [13]. One of the most effective data driven modeling techniques has been proven to be artificial neural networks (ANN)s [14]. In this regard, many recent applications of neural networks have emerged in literature for monitoring of strains and stresses during load cycles using strain gauges [15], pavement defect segmentation using a deep autoencoder [16], and machine learning based continuous deflection detection for bridges using fiber optic gyroscope [17].

In this work, an ANN based approach for realtime localization and the estimation of loads acting on aircraft wings from full field depth measurements is proposed. The proposed methodology can work with a single external depth image sensor with full field measurement capability for a single wing; thus, one sensor is enough for inspection of the whole wing. Moreover, depth cameras do not require any calibration and can be directly used on any kind of wing regardless if it was made of composites or not due to optical measurement. The proposed framework is able to estimate the magnitude of the load causing wing deflections under both bending and twisting loading conditions; therefore, it is not limited to pure bending case, as was in the work of Nicolas et al. [11]. Moreover, the proposed method is not just limited to the estimation of load magnitudes, but it will also be able to estimate the location of the load causing bending and twisting deflections; therefore, making the localization of the loads possible. The localization of loads can become a very useful tool, especially in the case when one needs to know the nature of the external loads occurring in flight. Using this information, one can estimate the exact flight conditions and, as such, can improve the design of the aircraft based on this new data. More importantly, the proposed framework can operate in real time. To the best of the authors’ knowledge, this is the first work in the literature to address the problems of real time localization and estimation of bending and twisting loads causing deflections to structures based on depth imaging and ANN.

The rest of the paper is structured, as follows; in Section 2, the proposed method for real time load monitoring from depth measurements using neural networks is presented. In Section 3, the experimental setup, data collection procedure, and evaluation of the used depth sensor for load monitoring are described. The effectiveness of the proposed approach is validated by an experimental study in Section 4, followed by the conclusion in Section 5.

## 2. A Data Driven Methodology for Realtime Monitoring of Loads from Depth Images

This work proposes the development of a data driven modeling approach for localization and estimation of loads acting on aircraft wings from full field depth measurements. These measurements can be provided by a multitude of sensors, such as depth cameras. An autoencoder coupled with two different supervised ANN architectures are proposed for the localization and estimation of loads in order to develop these models and ensure realtime performance. The localization part is treated as a multinomial logistic classification problem and the load magnitude estimation as a logistic regression problem, which are explained in detail in the following subsections.

### 2.1. Dimensionality Reduction Using Autoencoders

To develop data driven models for localization and estimation of loads from depth measurements while providing realtime performance, an autoencoder [18] framework is proposed to be utilized. This is because the full field measurements that are acquired from the depth sensors are inherently rich, but can be very large in size, thus working with them becomes computationally expensive and can hinder realtime performance. Autoencoders can effectively reduce the large number of features obtained from depth sensors while retaining the critical information, thus encoding the original input at a much smaller dimension. Furthermore, to ensure that maximum informative data is obtained, Kullback-Leibler divergence ($KL_{Div}$) [19] was used to avoid obtaining binary encoded data by enforcing the mean and standard deviation of the encoded data to be some desired values. In this work, logarithmic normalization was utilized to minimize this large range of data due to the possible presence of a large gap between the values of the input depth measurements. The overall algorithm for the utilized autoencoder is given, as follows:

(1) $$Y=\Gamma (<\log\left(X\right),W_{1}>+B_{1})$$

(2) $$Z=\Gamma (<Y,W_{2}>+B_{2})$$

(3) $$KL_{Div}=\alpha_{d}\log\frac{\alpha_{d}}{\alpha }+\left(1-\alpha_{d}\right)\log\frac{1-\alpha_{d}}{1-\alpha }$$

(4) $$CF_{AE}=\frac{1}{N}\sum_{i=1}^{N}{\left(X_{i}-Z_{i}\right)}^{2}+\beta KL_{Div}$$

where *X* is the input depth vector, *Y* is the output of the encoder, *Z* is the output of the decoder, Γ is the activation function, $\alpha_{d}$, and α are the desired and actual mean and/or standard deviation of the encoded data, respectively, $W_{1}$ and $W_{2}$ are the weight matrices, $B_{1}$ and $B_{2}$ are the bias vectors, $CF_{AE}$ is the autoencoder cost function to be minimized, and <·,·> is the dot product.

**Figure 1:**
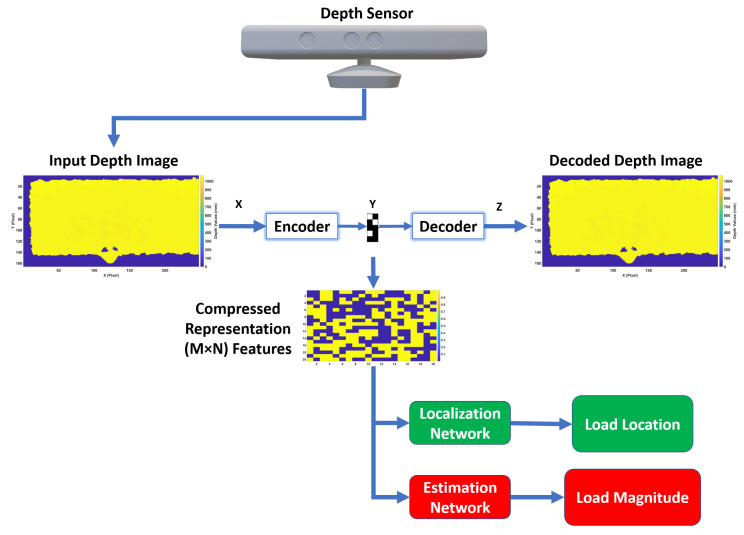
Proposed realtime load localization and estimation framework for SHM.

After the critical information is extracted from the input depth features and is encoded at a smaller dimension using the proposed autoencoder, two different supervised ANNs for realtime localization and estimation of loads can then be utilized, as illustrated in Figure 1.

### 2.2. Load Localization from Depth Images Using ANN

**Figure 2:**
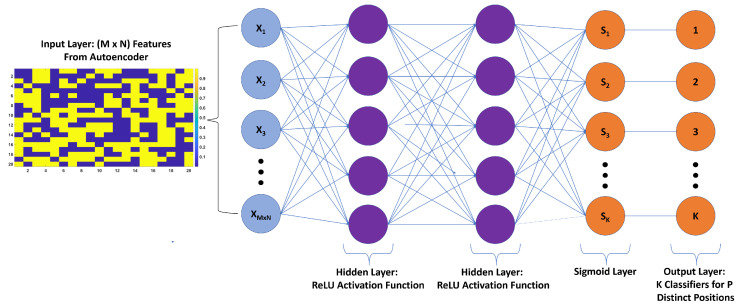
The proposed load localization ANN with 2 hidden layers and ReLU activation functions.

A supervised feed forward ANN with a classification structure is proposed to localize where the load is acting on the wing. The proposed ANN classifier, as depicted in Figure 2, takes the encoded depth images as inputs and provides output based on the location of the loads.

The encoded depth features for each sample in the training set were normalized across the samples by making use of the standardization technique so that the input features had zero mean and unit standard deviation. The formula used for standardization is given by Equation (5). As for the test set, they were standardized using the mean and standard deviation of the training set. Standardization was performed due to inevitable sensor noise, which can hinder the generalization capabilities of neural networks. Afterwards, the standardized inputs were fed into the localization ANN consisting of two hidden layers. The activation functions in both layers were chosen to be ReLU (Rectified Linear Unit), among other functions, such as sigmoid and tanh, due to ReLU’s fast convergence.

(5) $${\hat{X}}_{i}=\frac{X_{i}-mean\left(X_{j}\right)}{\sigma (X_{j})}$$

where $X_{i}$ vector contains the encoded features in each sample, $X_{j}$ vector contains the features across the samples, ${\hat{X}}_{i}$ vector contains the standardized features for each sample, and σ is the standard deviation.

Typically, in classification problems, the output labels are one hot encoded, through which, categorical data, in this case, the load positions, are converted into numerical data. The output of the last layer of the neural network, which is now a one hot encoded vector is passed through a sigmoid function. The Sigmoid function changes the arbitrary output scores to a range of probabilities that range between zero and one. Sigmoid, instead of other activation functions, was chosen to be in the output layer. This is because the load localization in this work is a multi label classification problem where more than one correct label exists in the output. Therefore, the output labels are not mutually exclusive i.e., the output labels are independent. The closeness between the output of the sigmoid function and the true labels (*T*) is defined as loss or cost function. The cost function of the classification ($CF_{CL}$) is defined as the average of Cross Entropy Error Function (CEEF) over a batch of multiple samples of size *N* and labels of size *K*, as follows:

(6) $$CF_{CL}=\frac{1}{N}\sum_{i=1}^{N}\sum_{j=1}^{K}T_{ij}\log\left(S\left(x_{ij}\right)\right)$$

The optimizer in the backpropagation algorithm updates the weights and biases, so as to minimize this loss and, as such, the loss decreases and the accuracy of the neural network increases. A classification ANN with two hidden layers of ReLU activation functions was determined to be sufficient to successfully localize the loads causing bending and/or twisting deflections. The proposed ANN was trained using Adam [20] optimizer. Both L2 regularization and dropout [21] techniques were utilized in order to increase the generalization performance of the network and prevent overfitting. This resulted in obtaining a new cost function, which consists of both the cost function defined by Equation (6) and the new scalar regularization value β due to L2 regularization. The final cost function $FCF_{CL}$ is given by Equation (7) and the metric used for calculating the accuracy of predictions in load localization is given by Equation (8). The localization results obtained for both concentrated and distributed loading scenarios are presented and evaluated in detail in the results section.

(7) $$FCF_{CL}=CF_{CL}+{\beta \sum ||Weights||}_{2}$$

(8) $$Accuracy_{CL}=\frac{\sum (Y=\hat{Y})}{N}\times 100$$

where *Y* is the ground truth, $\hat{Y}$ is the prediction, and *N* is the number of samples.

### 2.3. Load Estimation from Depth Images Using ANN

**Figure 3:**
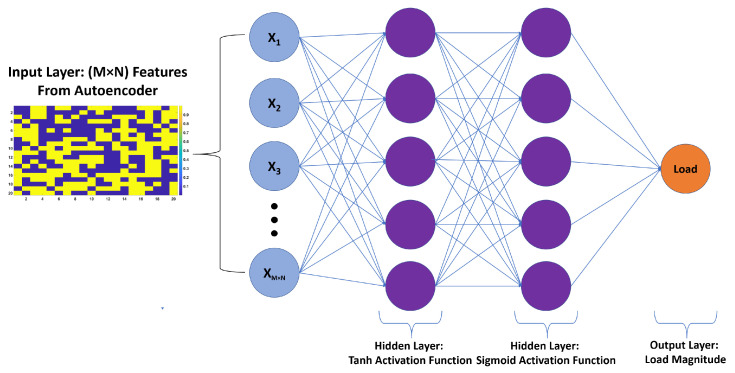
The proposed artificial neural networks (ANN) for estimation of loads with two hidden layers of tanh and sigmoid activation functions, respectively.

In this section, a logistic regression ANN for the estimation of the magnitude of loads acting on the wing is proposed. The input to this network is again the encoded depth images, and the output is the magnitude of the load. Unlike the ANN classifier, the output layer here consists of only a single node which provides continuous type numeric outputs in terms of loads. Because the output, in this case, is a single numeric value, there is no need to use sigmoid function in the output, as was the case in logistic classification. Moreover, the cost function for the estimation of load magnitudes ($CF_{E}$) is simply defined as the sum of the squared difference between the predicted value and the ground truth as given by Equation (9). Similar to the localization part, the estimation of load magnitudes was performed using two hidden layers, but the activation functions used in the first and second hidden layers were chosen to be tanh and sigmoid, respectively. The proposed load estimation ANN is illustrated in Figure 3.

(9) $$CF_{E}=\frac{1}{N}\sum_{i=1}^{N}{\left(Y-\hat{Y}\right)}^{2}$$

where *Y* is the ground truth, $\hat{Y}$ is the prediction, and *N* is the number of samples.

Both tanh and sigmoid functions belong to the family of sigmoid functions. The difference between these two is that the output of sigmoid function ranges from zero to one while the output of tanh ranges from −1 to +1. Moreover, the tanh function often converges faster than sigmoid due to tanh’s symmetric nature [22]. The formula used for calculating the accuracy of predictions [23,24] for load estimation is given by Equation (10). The results obtained for both concentrated and distributed loading scenarios are presented and evaluated in detail in the experimental results section.

(10) $$Accuracy_{E}=\left(1-\frac{∥Y-\hat{Y}∥}{∥Y-\bar{Y}∥}\right)\times 100$$

where *Y* is the ground truth, $\hat{Y}$ is the prediction, and $\bar{Y}$ is the mean of the ground truth.

## 3. Experimental Setup and Evaluation of the Depth Sensor for Load Monitoring

### 3.1. Experimental Setup

**Figure 4:**
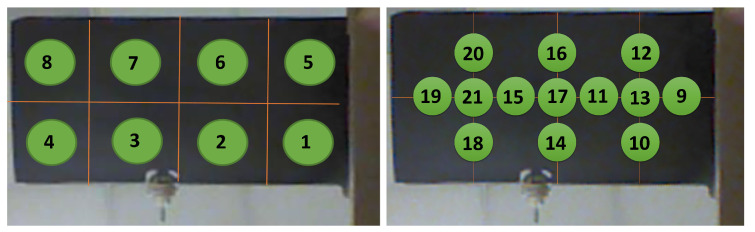
Positions of concentrated (**Left**) and distributed (**Right**) loads (Green) acting on the UAV wing.

In order to validate the effectiveness of the proposed framework, an experimental setup that consists of a composite wing of a quad tilt-wing aircraft called SUAVI [25] was used. The wing has a size of 50 × 25 cm in length and width, respectively. The root side of the wing was fixed, so that no tilting was induced under applied loads. Similar to the works in the literature, ground tests were performed to experimentally mimic the deflections that may occur on a wing due to some external loads during flight. In the experiment, different types of loads that cause bending and twisting deflections on the wing were applied in two different loading scenarios. First, the load was designed to be acting on one of the eight positions depicted in the left image of Figure 4 and it is called concentrated loading case in this work. Six calibrated loads with magnitudes of [2.45, 4.9, 7.35, 9.81, 12.26, 14.71] N were chosen to be acting on these positions. Therefore, in total, eight different positions exist with each one containing six distinct loads, resulting in forty eight configurations for the first case. In the second scenario, the loads were chosen to be distributed loads placed in between each of the eight locations. This way the loads were made to be acting on multiple locations of the wing at the same time, as indicated in the right image of Figure 4. In loading positions 9 to 21, except for positions 13, 17, and 21, the loads were made to act at two positions simultaneously, for example position 9 represents two loads acting at positions 1 and 5. As for positions 13, 17, and 21, the loads were made to act at four positions simultaneously, for example position 13 represents four loads acting at positions 1, 2, 5, and 6 at the same time. Therefore, the total number of positions corresponding to distributed loads are thirteen. The magnitude of loads used for distributed scenario were the same as the concentrated loading case, but their magnitudes were distributed among the multiple positions they were acting upon, for example at position 9 two loads of 1.225 N were acting at positions 1 and 5 simultaneously for a loading case of 2.45 N. Therefore, six distinct load magnitudes per location exist in the distributed loading case, thus resulting in seventy eight different configurations for the second case. Therefore, in total, 126 distinct loading cases were performed during the experiments. In order to measure the deflections occurring over the span of the wing, this work proposes the use of a single RGB-D camera. RGB-D cameras are sensors that are capable of providing pixel wise depth information from images, thus making them suitable for full field measurement purposes. The RGB-D sensor used for data collection in this work was chosen to be a Microsoft Kinect V1 [26] sensor. The reasons for choosing Microsoft Kinect V1 for this work are as follows:

- It has high resolution depth and visual (RGB) sensing and is offered at a very affordable price when compared to other three-dimensional (3D) cameras, such as SwissRanger [27] and other Time of Flight cameras [28].
- It works based on structured light thus making it suitable for measurement from an inclined angle.
- It works in realtime (@ 30 Hz) with a field of view (FOV) of $43^{\circ }\;$ (vertical) × $57^{\circ }\;$ (horizontal) and can measure an area of 1.5×1 m from a distance of 1 m.
- One of the advantages of using a depth camera like Microsoft Kinect is that it does not require the sophisticated and time consuming camera calibration procedures since it is already calibrated, and it directly provides X, Y, and Z information in the camera frame and without loss of generality this frame is also the world frame.
- Moreover, depth cameras have many advantages over conventional intensity sensors in that they can work in low light conditions and are invariant to texture and color changes [29].

**Figure 5:**
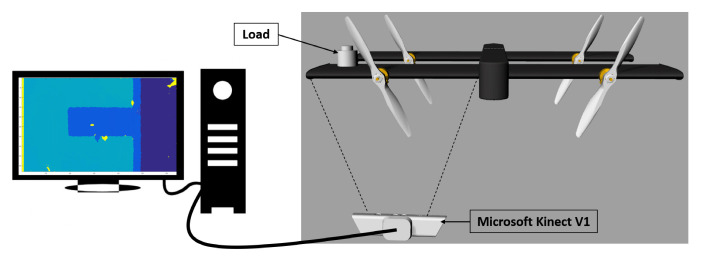
Experimental setup.

**Figure 6:**
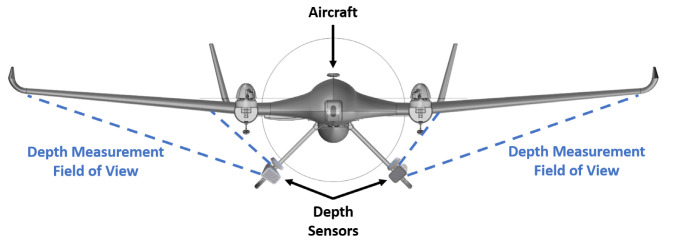
Possible installation locations of depth sensors on large aircraft.

Khoshelham et al. [30] theoretically and experimentally evaluated the geometric quality of the depth data that were collected by Kinect V1. They quantified the random error of depth measurement to be 4 cm at a ranging distance of 5 m, and concluded that the error increased quadratically by the increase in the ranging distance from Kinect. Based on their results, Khoshelham et al. made a recommendation for the use of Kinect sensor for general mapping applications, and they indicated that the data should be acquired at a distance of 1 to 3 m from the sensor due to the reduced quality of range data at further distances. Therefore, in this work, the Kinect was placed at a distance of one meter from the wing during the tests, and it was placed under the wing of the UAV, so as to capture the whole wing. Figure 5 shows the experimental setup used in this work. Although the methodology will be evaluated for a relatively small aircraft, the same technique can be utilized for structural health monitoring of much larger ones with the use of a depth camera with larger field of view, such as Carnegie Robotics^®^ MultiSense™ S21B [31], Arcure Omega [32], and MYNT EYE [33]. Moreover, because the UAV used in this work was small in size, the depth sensor was not installed on it. Nonetheless, depth cameras can be installed on a larger aircraft with a minimum distance according to the depth sensor’s operation range between the wing and the installation location. Depth cameras could be installed in place of RGB cameras that were fitted on the aircraft [3,4,7] but with the advantage of not requiring installation of additional marker’s or LEDs on the wing as shown in Figure 6. The installation of the depth sensors at an angle, as shown in Figure 6, will not affect their operation, since, once a deflection occurs over the span of the wing, the wing’s depth will change with respect to the pose of the installed depth sensor. For large aircrafts, the depth sensors can be connected to an onboard pc via wired or wireless connections. Moreover, dampers can be utilized for reducing the impact of vibrations on the depth sensors in order to take into account the vibrations that may occur in flight. Furthermore, if the proposed method is trained with the data obtained from in flight conditions then the proposed method can take into account all of the disturbances acting on the depth camera, since the disturbances will manifest themselves in the acquired data.

### 3.2. Data Collection Procedure

**Figure 7:**
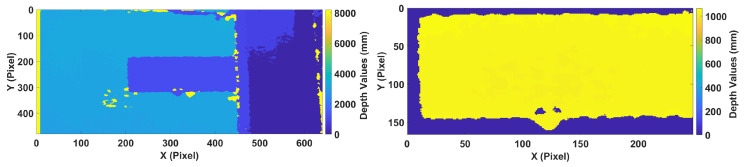
(**Left**) Acquired and (**Right**) Extracted depth image of the composite UAV wing.

The data collection procedure was performed similar to the works in the literature [1,2,3]. For both concentrated and distributed loading scenarios, first, loads were applied at each position as depicted in Figure 4 separately, and then depth images of the wing were acquired. For example, a load of 2.45 N was applied on position 1, while no other load was applied at any other location, and then the data was collected. Afterward, the next load of 4.9 N was applied at the same position, and the data were recorded. This procedure was repeated for all other locations in the same manner until data from all of the positions with all of the different load magnitudes were recorded. The acquired depth images by Kinect V1 are shown in Figure 7, in which the pixel values correspond to the actual measured distance in mm. Because the images were captured at one meter distance with a resolution of 640×480 pixels, the captured scene encompassed much more information than required; therefore, the images were cropped to include only the UAV wing, and the size of the acquired image was reduced to 247×166 pixels. It should be noted that this was only done in this case and, if the whole wing encompasses the image, then there is no need for cropping the image. Moreover, any depth values above 2000 mm and below 800 mm were changed to zero in order to get rid of redundancies, as shown in the right image of Figure 7. Moreover, since the gap between the depth values of the wing and its surrounding were very large, the color distribution resulted in a binarized representation.

### 3.3. Evaluating Accuracy and Precision of Microsoft Kinect V1

**Figure 8:**
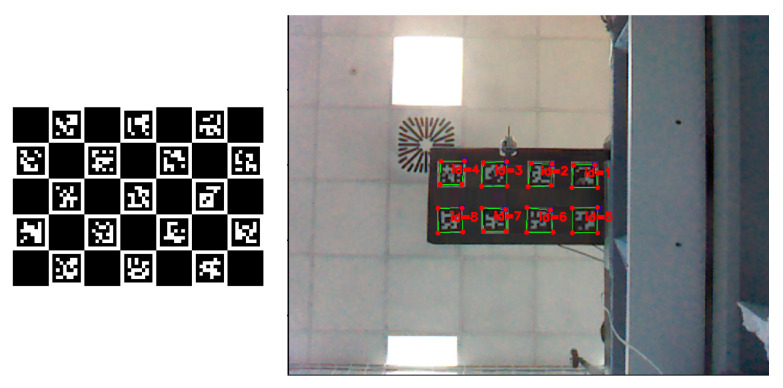
(**Left**) Sample ArUco Markers. (**Right**) Corner extraction in subpixel accuracy from ArUco markers.

Even though Microsoft Kinect V1’s overall accuracy and precision were previously evaluated by Khoshelham et al. [30]; in this work, its evaluation was performed for the specific working conditions required for structural health monitoring. To this end, the deflections at 32 reference points of the wing were measured using Kinect V1 sensor, and they were compared with the measurements from a laser ranger. To track the locations of reference points in the image plane, eight ArUco [34] markers, each having four corners were used. The patterns known as ArUco markers are small two-dimensional (2D) barcodes often used in augmented reality and robotics, as shown in Figure 8. ArUco was developed by Garrido-Jurado et al. [34], where they showed the superiority of their work to other known markers in literature such as ARTOOLKIT [35], ARToolKit Plus [36], and ARtag [37]. The locations of these 32 corners in image plane were detected in subpixel accuracy using the algorithm provided by Garrido-Jurado et al. [34] and the obtained results are shown in Figure 8.

#### 3.3.1. Mapping Depth and RGB Images of Microsoft Kinect V1

To use ArUco markers for tracking the corners during deflection measurements, the RGB and depth images obtained from Kinect V1 were mapped using an affine transformation. Using *n* known points of the wing, such as its four corners, the unknown parameters of the transformation can be calculated, as follows:

(11) C=AD

(12) $$A=CD^{-1}$$

where *C* ∈${ℜ}^{2\times n}$ contains the locations of a point in the colored image, *D* $\in {ℜ}^{3\times n}$ contains the corresponding locations in the depth image, and *A* ∈${ℜ}^{2\times 3}$ is the affine transformation matrix.

**Figure 9:**
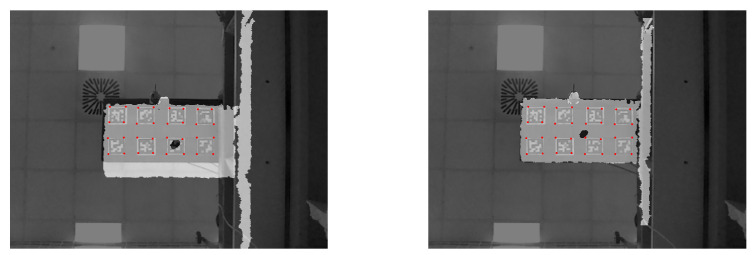
Kinect V1 depth and RGB images in grayscale (**Left**) Before mapping. (**Right**) After mapping.

By following the above steps, depth images were successfully mapped to the RGB images that were acquired from the Kinect V1, as shown in Figure 9. As seen from this figure, the color images were changed to grayscale, so as not to work with unnecessary color channels. This way, the corners of the ArUco markers, which act as reference points, can be used for tracking the deflection changes at these points both by Kinect V1 and a laser meter, which will act as ground truth.

#### 3.3.2. Microsoft Kinect V1 vs Leica DISTO X310 Laser Meter

**Figure 10:**
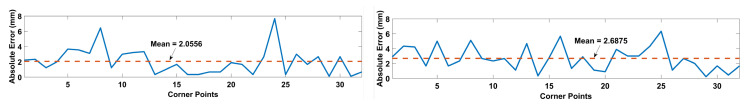
Absolute errors at 32 corner points of the wing for (**Left**) 2.45 N load. (**Right**) 14.71 N load.

**Figure 11:**
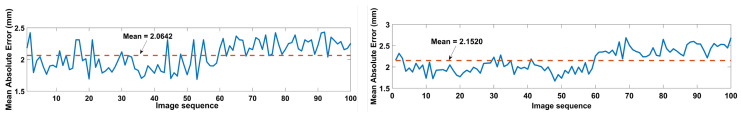
Mean of absolute errors in 100 frames for (**Left**) 2.45 N load. (**Right**) 14.71 N load.

After the RGB and Depth images were mapped, the difference between the deflections that were measured by Kinect V1 and laser ranger at corner points were calculated. To account for errors in corner detection and mapping, for each detected corner pixel from RGB image, a 3×3 window of pixels was chosen from the corresponding depth image. A Leica DISTO X310 laser meter [38] with ±1 mm accuracy was used for evaluating the accuracy of Kinect V1. Loads with magnitudes of 2.45, 4.9, 7.35, 9.81, 12.26, and 14.71 N were placed on position 4 of the wing and the deflections were measured using both the Kinect V1 and Leica DX310 sensors. The obtained results are shown in the left image of Figure 10 for 2.45 N load, in which the mean absolute error for the 32 corner points was measured to be 2.0556 mm with a standard deviation of 1.74 mm. This was repeated for 100 frames, and the obtained results are shown in the left image of Figure 11, in which the mean of absolute errors was measured to be 2.0642 mm with a standard deviation of 0.21 mm. Similarly, the results obtained for 14.71 N load are shown in right images of Figure 10 and Figure 11, in which the mean absolute error for 32 corner points was calculated to be 2.6875 mm and 2.1520 mm for a single and 100 frames, respectively, with standard deviations of 1.615 mm and 0.26 mm. The same procedure was repeated for all of the aforementioned loads that range from 2.45 to 14.71 N, and the obtained results are summarized in Table 1. These results show that for the required working conditions, the Kinect V1 has an accuracy of ±2.25 mm with a standard deviation of 0.28 mm and a resolution of 1 mm. Therefore, since the deflections due to different loads range from 1 mm to 19 mm in this work, the Kinect V1 is proved to be suitable for acquiring the deflection measurements reliably. Moreover, neural networks already take the noise of the sensors into account when they are trained with the data acquired from them; therefore, the small noises in the acquired data are taken care of by the proposed method.

### 3.4. Visualization of Depth Features Acquired from Microsoft Kinect V1

**Figure 12:**
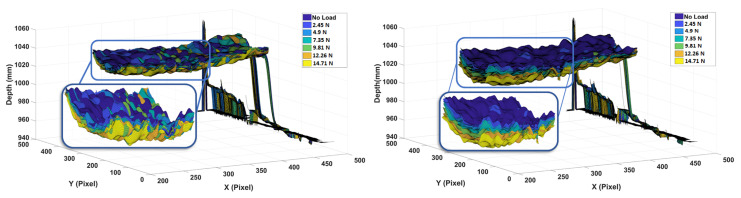
Wing deflections due to loads placed at (**Left**) Position 1 (**Right**) Position 8.

Here, the visualization of depth features that were obtained from Kinect V1 for loads acting on the wing is shown. As mentioned, the spanwise deflections of the wing due to the application of the external loads were captured by the depth camera. Figure 12 illustrates these deflection measurements for the distinct loads acting on position 1 and 8. These illustrations were made by reconstructing the surface of the wing from the acquired depth images after the application of each load. These images show how the overall bending profile of the wing changes due to the magnitudes and locations of the external loads. The Kinect V1 sensor can measure deflections due to loads as small as 2.45 N acting on position 1 of the wing, as seen from these measurements. Deflection profile is especially visible for loads acting on Position 8, since it is the furthest point from the fixed part of the wing.

**Figure 13:**
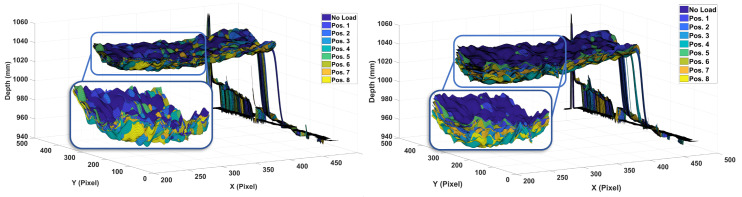
Wing deflections due to (**Left**) 2.45 N and (**Right**) 14.71 N loads applied on distinct positions of the wing.

Moreover, in order to show the effect of the same load applied on different sections of the wing, loads of 2.45 and 14.71 N were applied on all eight distinct partitions of the wing, and the resulting depth images were plotted in Figure 13. These results clearly indicate that the wing deflection profile is different even when the same load is applied on different locations. Furthermore, by observing these results one can see a strong relationship between the behavior of the deflection of the wing for loads placed in parallel, namely loads placed at Positions 1 and 5, 2 and 6, 3 and 6, 4 and 8. These results prove the effectiveness of Microsoft Kinect V1 sensor for wing deflection measurement under various loading conditions acting on distinct sections of the wing.

## 4. Dataset Creation and Experimental Results

### 4.1. Dataset Creation

A dataset of wing deflections due to loads was constructed by extracting the depth features from the Microsoft Kinect V1 sensor operating at 30 Hz by following the procedure explained in Section 3.2 in order to train and test the proposed ANN models. To construct the training dataset, 100 samples for each loading case was acquired. Initially, depth images were acquired when no load was acting on the wing. Afterward, data were collected for six different load magnitudes acting on 21 distinct sections of the wing in sequence, which included the concentrated and distributed loading scenarios. Therefore, 12,700 samples were collected for the training dataset. As for the test dataset, the depth images were acquired for the duration of only one second, thus resulting in 30 samples for each loading case for this dataset. Unlike the training dataset, the six distinct loads on the 21 sections of the wing were applied at random in this case, so as to evaluate the robustness of the proposed approach. This resulted in the acquisition of 3810 samples for the test dataset. The details of the constructed training and test datasets are tabulated in Table 2.

In this paper, TensorFlow [39] software was used to build and test the proposed ANN models. TensorFlow is an open source platform developed by Google Brain team, and it is widely used in literature to conduct machine learning based research due to its highly efficient computation framework. The computer used for developing the proposed ANN models had an Intel Xeon 3.6 GHz, twelve thread central processing unit (CPU) with 16 GB of RAM, and the whole network was trained on the CPU only, without the need of GPU.

### 4.2. Experimental Results and Discussions

In this section, the performance of the proposed framework for load localization and estimation are analyzed and discussed in detail. Initially, autoencoders were used to extract informative data from the depth images at a much smaller scale. subsequently, the proposed ANNs were trained using the training set described in Section 4.1, and their performance was evaluated and compared with a modified version of Castigliano’s theorem (The readers can refer to the Appendix A for the details of this algorithm.) on the constructed test set.

#### 4.2.1. Load Localization From Depth Images Using ANN

An autoencoder was used to obtain informative data at a much smaller scale from the acquired depth images to be fed into the localization and estimation networks, as stated in Section 2.1. The autoencoder was run in series with the classification network so as to use its accuracy as a measure of performance in order to determine the smallest size of the encoded features required for load localization. The encoded feature size was initially set to be 400 and then was increased with increments of 200 until satisfactory load localization performance was obtained. As described in Section 3.1, two different loading scenarios were considered in this work. In the concentrated loading scenario, the output locations were labeled positions 1 to 8 and, in the distributed loading scenario, they were labeled positions 9 to 21. Moreover, 0 label was chosen to represent the no load condition. Therefore, the total number of distinct positions in the output layer amounts to 22. By making use of one hot encoding technique, the aforementioned 22 distinct positions were encoded using only nine classifiers in the output of the localization network by setting *K* to 9 and *P* to 22. Labels 1 to 21 were one hot encoded using the first eight classifiers, and the no load case was encoded using the ninth classifier.

The proposed autoencoder and classification network was trained by varying the encoded data size. The activation function of the autoencoder was chosen to be a sigmoid and the number of neurons in the first and second hidden layers of the classification network were set to 60% and 30% of the encoded data size, respectively. The mean and standard deviation of the encoded data to be used in Equation (3) were chosen as 0.5 and 0.2, respectively. The β coefficient for regularization was chosen to be 0.1, and the dropout ratio of 0.8 was chosen in order to increase the network’s generalization capabilities. Moreover, the starting learning rate was chosen to be 0.0005 and the network was trained using Adam optimizer. The training of the network was performed for 8000 iterations for each encoded data size, and the results are tabulated in Table 3. From these results, it is observed that, as the size of encoded data increases, the difference between the training and test accuracies decrease. This suggests that more distinctive data is being extracted as encoded data size increases. Moreover, an encoded data size of only 1200 was enough for obtaining very high accuracies of 96.4% and 94.3% when evaluated on the training and test datasets, respectively.

**Figure 14:**
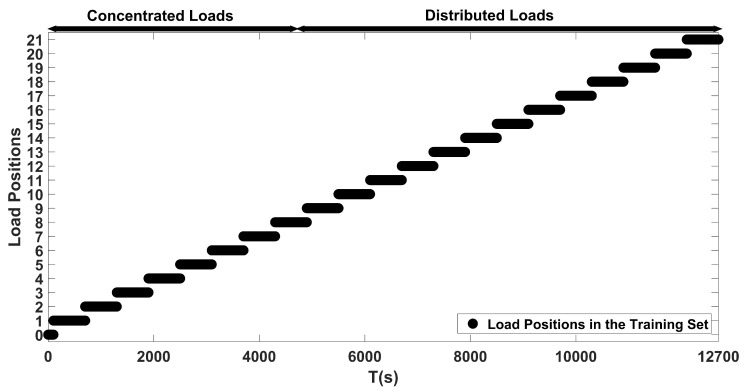
Load positions used for training the proposed localization ANN.

**Figure 15:**
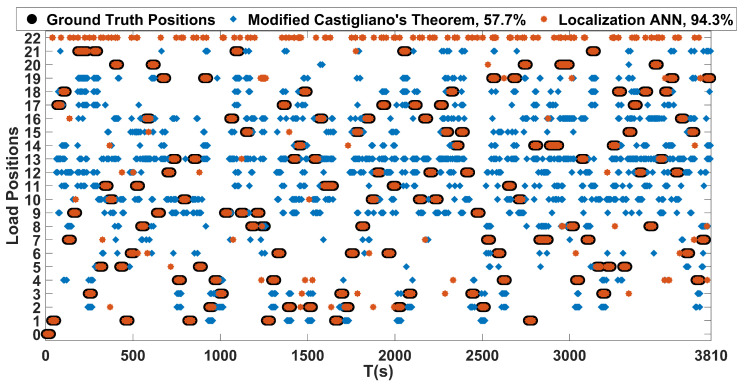
Position predictions based on the proposed localization ANN and modified Castigliano’s theorem, evaluated on the test dataset.

As for load localization that is based on the modified Castigliano’s theorem, its performance was also evaluated on the constructed test dataset. The proposed localization ANN was trained with the load locations shown in Figure 14. Outputs of the proposed localization network with an encoded data size of 1200 and the one based on Castigliano’s theorem are plotted against the ground truth positions in Figure 15. In this figure, the label denoted as zero represents the no load case, labels 1 to 8 represent the concentrated load positions, labels 9 to 21 represent the distributed load positions, and label 22 represents misclassified outputs that do not belong to any of the aforementioned load positions. From these results, one can see that both frameworks are able to discern the locations of the loads causing different kinds of bending and twisting deflections on the wing rather successfully. However, the accuracy of the proposed ANN based framework is superior to the one that is based on Castigliano’s theorem, as visible from the results obtained in Figure 15 and the obtained accuracies given in Table 4. More importantly, the proposed neural network based method exhibits invariance to the type of the applied load and is able to successfully localize both concentrated and distributed loads causing wing deflections, which the framework based on modified Castigliano’s theorem fails to do so properly.

**Figure 16:**
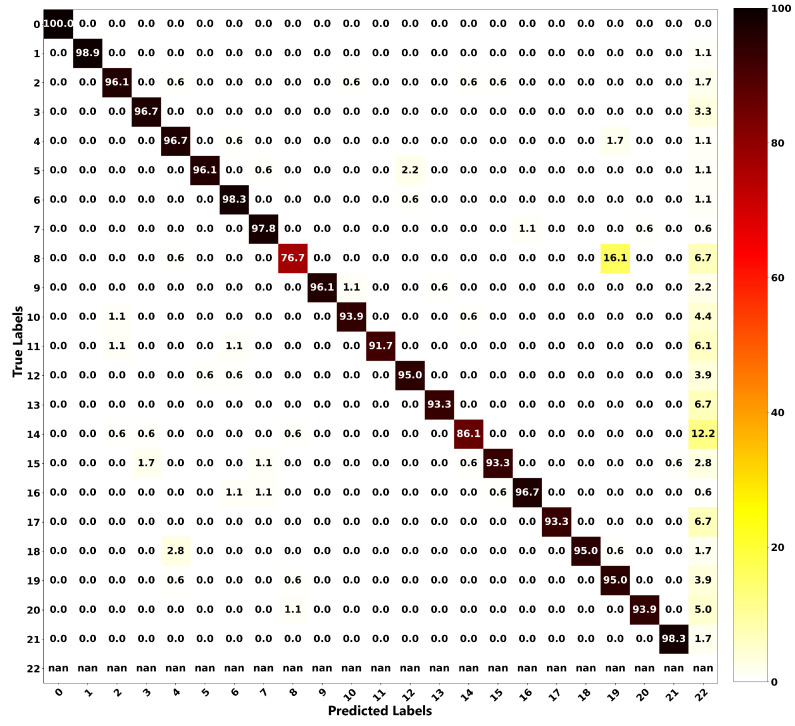
Confusion matrix for localization based on the proposed ANN, evaluated on test dataset.

**Figure 17:**
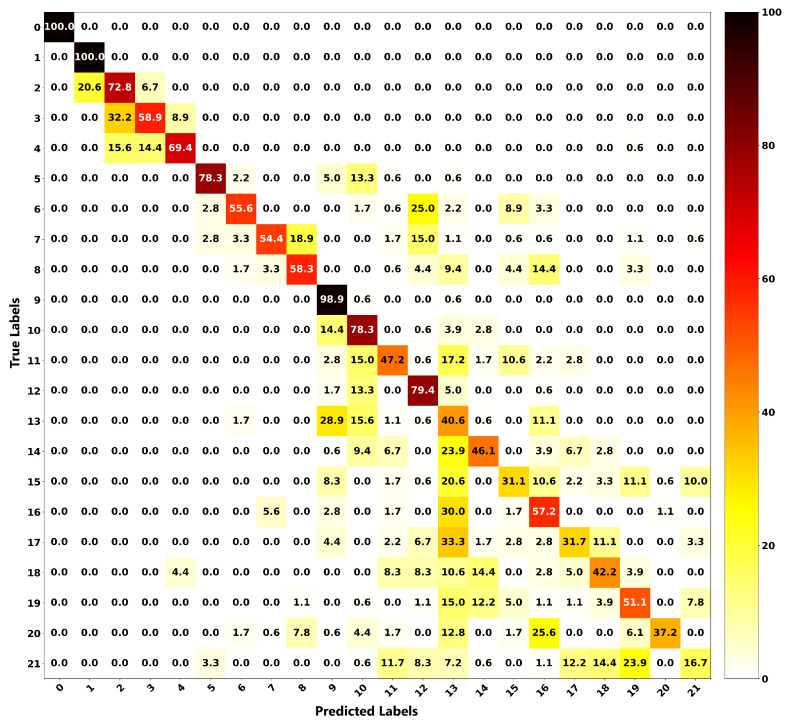
Confusion matrix for localization based on modified Castigliano’s theorem, evaluated on test dataset.

The relationship between the ground truth and the estimations of both frameworks is illustrated as a confusion matrix in order to get more insight into the localization performance of both methods. Figure 16 shows the confusion matrix for the proposed localization ANN, and Figure 17 is for the modified Castigliano’s theorem. From these plots, it can be seen that the correct estimations are on the diagonal and any points not located on this line represent misclassified outputs. For the ANN framework, all of the position estimations have accuracies of higher than 90%, except for Positions 8 and 14. Nonetheless, their accuracies are still high, being 76.7% and 86.1%, respectively. As observed from Figure 16, Position 8, which is a concentrated load, is misclassified 16.1% of the time as Position 19, which is a distributed load. This observation is due to the wing deflection profiles under these loading conditions having similar patterns. However, these observations seem to be worse for the results obtained through the modified Castigliano’s theorem, especially in the case of distributed loads, as seen in Figure 17. This is due to the fact that measured deflections exhibit very similar patterns at different points and, therefore, can not be accurately captured unless a more robust model such as the proposed ANN one is used to classify them in an appropriate manner.

#### 4.2.2. Load Estimation from Depth Images Using ANN

In this subsection, the proposed autoencoder and regression ANN’s performance for load estimation is evaluated on the same dataset as the localization one. Unlike the localization networks’ output, the output of this network is a single continuous variable that represents the magnitude of applied load under both concentrated and distributed loading conditions. Similar to the localization part, the proposed load estimation network was trained with varying encoded data size and the same parameters of the autoencoder network. As for the regularization coefficient β and dropout rates, they were fine tuned to be 0.1 and 0.35, respectively. This time the training was performed for 30,000 iterations for each encoded data size, and the obtained results are shown in Table 5. Based on the obtained results, it is seen that, again, an encoded data size of 1200 neurons was enough for obtaining very high accuracies of 97.3% and 92.7% when evaluated on the training and test datasets, respectively. Besides, it is observed that training such a network with good performance requires significantly more iterations when compared with the classification one, since the output in regression problems is a continuous variable.

**Figure 18:**
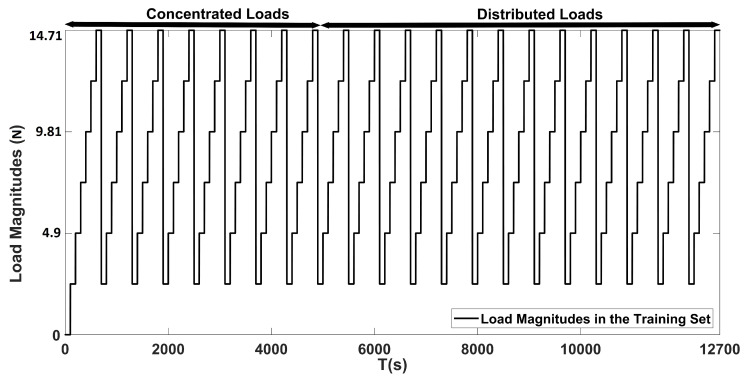
Load magnitudes used for training the proposed estimation ANN.

**Figure 19:**
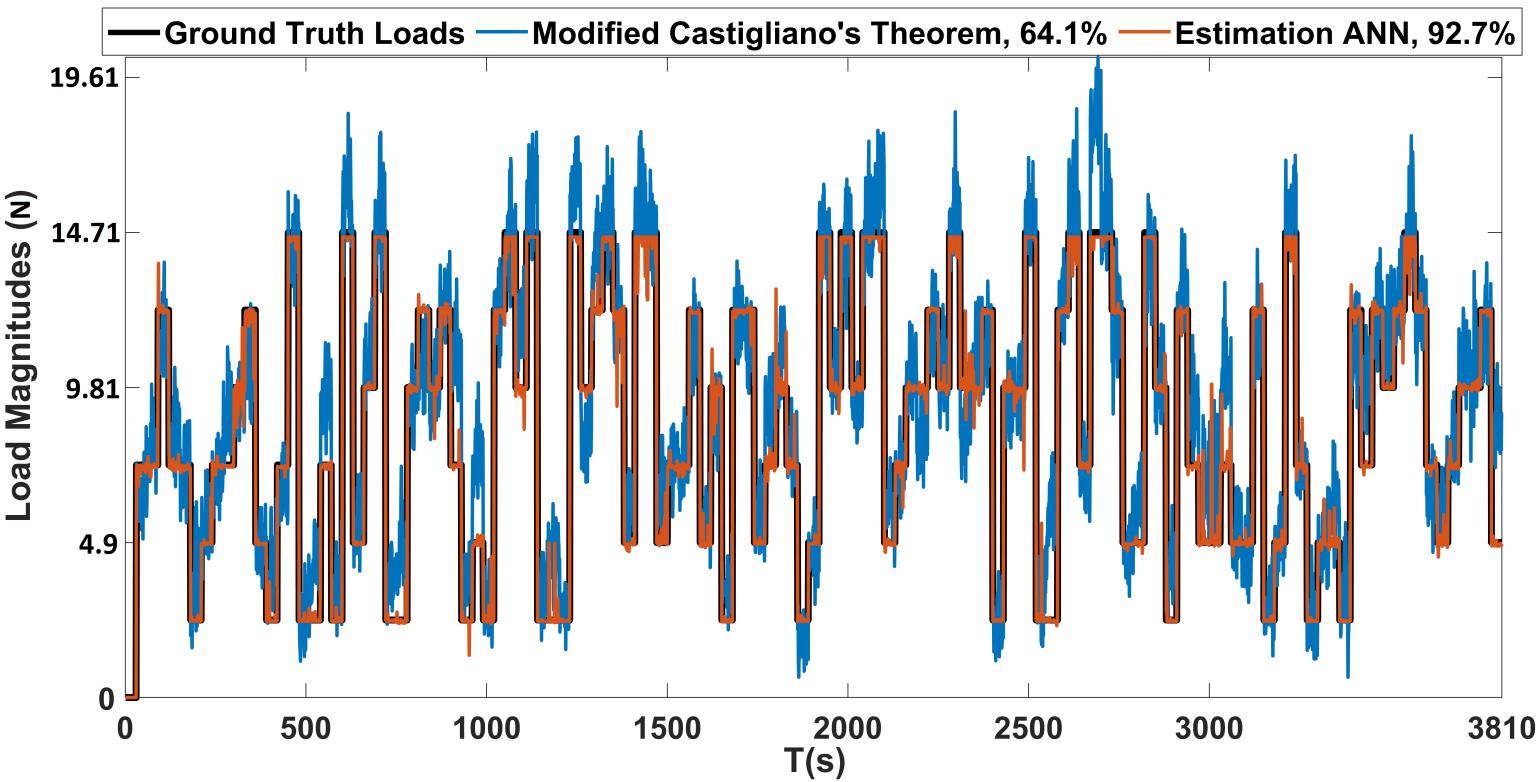
Load estimation based on the proposed ANN and modified Castigliano’s theorem, evaluated on test dataset.

Moreover, the performance of load estimation that is based on modified Castigliano’s theorem was also evaluated on the constructed test dataset. The proposed estimation ANN was trained with the loads that are shown in Figure 18. The outputs of the proposed load estimation ANN with 1200 encoded data size and the modified Castigliano’s theorem are plotted against the ground truth loads presented in Figure 19. These results show that magnitudes of loads causing bending and twisting deflections can be estimated with very high confidence, regardless of where the load is acting on the wing. However, the accuracy of the proposed ANN based framework is far more superior when compared with the one that is based on the modified Castigliano’s theorem, as visible from the results obtained in Figure 19 and the obtained accuracies tabulated in Table 6.

These results show that the proposed method can localize and estimate the loads acting on an aircraft wing with very high accuracies under both concentrated and distributed loading conditions. It should be noted that, since the proposed method was trained with elastic loading cases, it will only work with elastic loads. If the wing is damaged, then the relationship between the deflections and the load will change; therefore, the proposed method will not work unless it is trained for the damaged cases as well. Even though the training of the proposed networks is performed offline, the proposed method requires only 0.02 s for data reduction from a full frame image with 640×480= 307,200 pixel features using an autencoder with an encoded data size of 1200. As for data reduction from the cropped images used in this work that had 247×166= 41,002 pixel features, an autoencoder with an encoded data size of 1200 requires only 0.008 s. Another 0.001 second is required for localization or estimation of loads from the encoded data. Therefore, the proposed method can operate at 0.021 s or around 47 Hz for a single full frame image, thus realizing real time performance.

## 5. Conclusions

In this work, a robust structural health monitoring system based on depth imaging and artificial neural networks for localization and estimation of bending and twisting loads acting on an aircraft wing in real time is proposed. The proposed framework is based on the usage of depth images obtained from a depth camera as input features to an autoencoder and load location or magnitude as output labels of supervisory neural networks placed in series with the autoencoder. Initially, the Microsoft Kinect V1 depth sensor’s accuracy and precision were evaluated for monitoring of aircraft wings by making use of ArUco markers and a Leica DISTO X310 laser meter having an accuracy of ±1 mm. The Kinect V1 proved to be reliable for SHM purposes, since it provided full field measurements with accuracy and standard deviation of 2.25 mm and 0.28 mm, respectively, when compared with the single point measurements provided by the laser meter.

As for the proposed method, first, an ANN consisting of an autoencoder and two hidden layer classification network with ReLU activation functions was proposed for estimating the location of loads. Second, an autoencoder and logistic regression network of two hidden layers with tanh and sigmoid activation functions was proposed for estimating the magnitude of these loads. Both of the proposed networks were trained and validated on an experimental setup, in which the application of concentrated and distributed loads were applied on a composite UAV wing.

In addition, a comparison with an approach based on Castigliano’s theorem was performed, and the proposed method proved to have superior performance in terms of the localization and estimation of loads. The proposed localization and estimation ANNs achieved accuracies of 94.3% and 92.7%, while the framework based on Castigliano’s theorem achieved average accuracies of 57.7% and 64.1%, respectively, when both of the methods were evaluated on a dataset containing randomly applied concentrated and distributed loads. As demonstrated, the proposed ANN based framework can localize and estimate the magnitudes of loads acting on aircraft wings with very high accuracies from a single depth sensor in realtime.

In the near future, it is planned to extend the current study for the localization and estimation of highly dynamic loads on larger aircraft wings.

## Figures and Tables

**Table 1:** Accuracy and Precision of Microsoft Kinect V1.

Loads (N)	2.45	4.9	7.35	9.81	12.26	14.71	Average
Absolute mean error (mm)	2.06	2.18	2.3	2.59	2.21	2.15	2.25
Standard deviation (mm)	0.21	0.36	0.46	0.22	0.15	0.26	0.28

**Table 2:** Training and test datasets.

	No. of Samples per Load per Position	No. of Distinct Loads per Position	No. of Distinct Positions	Total No. of Samples	No. of Features per Sample
No Load	100 (30)	1	1	100 (30)	247×166 (41,002)
Concentrated Loads	100 (30)	6	8	4800 (1440)	247×166 (41,002)
Distributed Loads	100 (30)	6	13	7800 (2340)	247×166 (41,002)
Training Dataset	100	—	22	12,700	247×166 (41,002)
Test Dataset	30	—	22	3810	247×166 (41,002)

The ( ) show the number of samples in the test dataset.

**Table 3:** Accuracy of the proposed localization ANN with varying encoded data size.

Encoded Data Size	${CF}_{AE}$	Layer 1 Neurons	Layer 2 Neurons	Training ${\mathrm{Accuracy}}_{\mathrm{CL}}$ (%)	Test ${\mathrm{Accuracy}}_{\mathrm{CL}}$ (%)
400	31.76	240	120	66.1	60.3
600	31.58	360	180	93.9	88.8
800	31.05	480	240	95.1	91.3
1000	29.98	600	300	96.2	93.6
1200	29.44	720	360	96.4	94.3

**Table 4:** Localization performance of the proposed ANN and modified Castigliano’s theorem, evaluated on test dataset.

Localization Method	Accuracy (%)
Localization ANN	94.3
Modified Castigliano’s Theorem	57.7

**Table 5:** Accuracy of the proposed load estimation ANN with varying encoded data size.

Encoded Data Size	${\mathrm{CF}}_{\mathrm{AE}}$	Layer 1 Neurons	Layer 2 Neurons	Training ${\mathrm{Accuracy}}_{E}$ (%)	Test ${\mathrm{Accuracy}}_{E}$ (%)
400	31.76	240	120	93.9	83.1
600	31.58	360	180	96.0	88.3
800	31.05	480	240	96.7	90.8
1000	29.98	600	300	96.4	91.5
1200	29.44	720	360	97.3	92.7

**Table 6:** Load estimation performance of the proposed ANN and modified Castigliano’s theorem, evaluated on the test dataset.

Load Estimation Method	Accuracy (%)
Estimation ANN	92.7
Modified Castigliano’s Theorem	64.1

**Figure A1:**
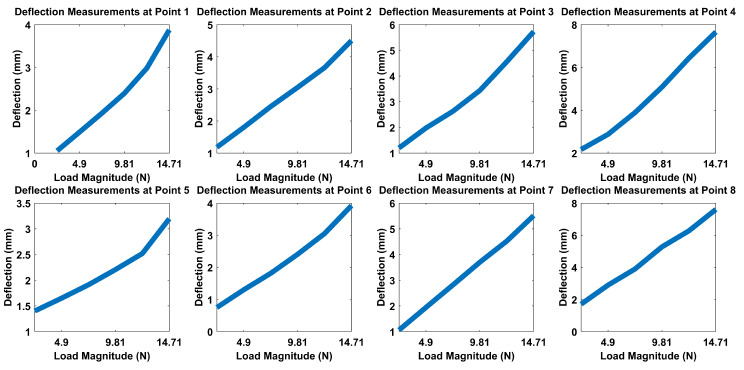
Deflection measurements for loads acting at point 2 of the wing.

**Figure A2:**
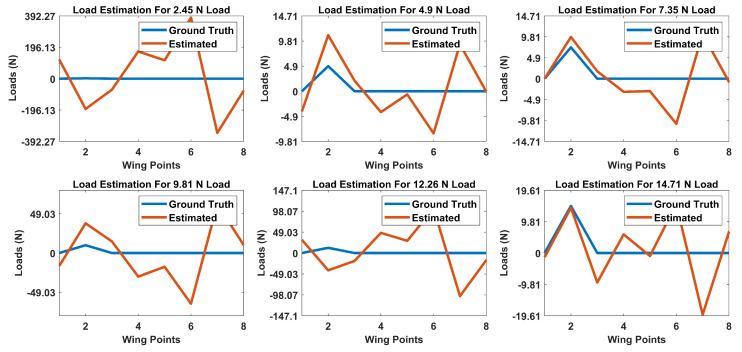
Load estimations for concentrated loads acting at point 2 of the wing.

**Figure A3:**
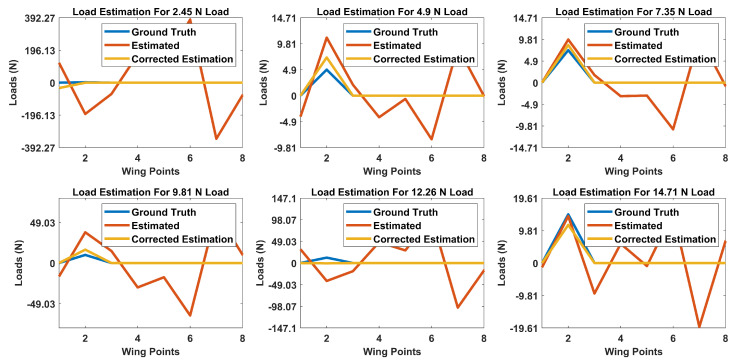
Corrected load estimations for concentrated loads acting at point 2 of the wing.

**Figure A4:**
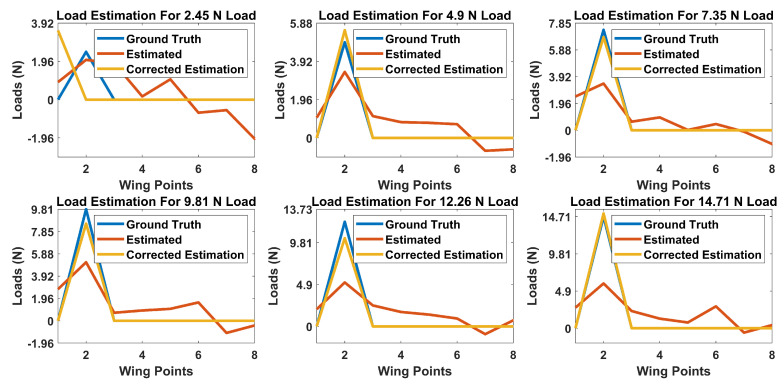
Optimized and corrected load estimations for concentrated loads acting at point 2 of the wing.

**Figure A5:**
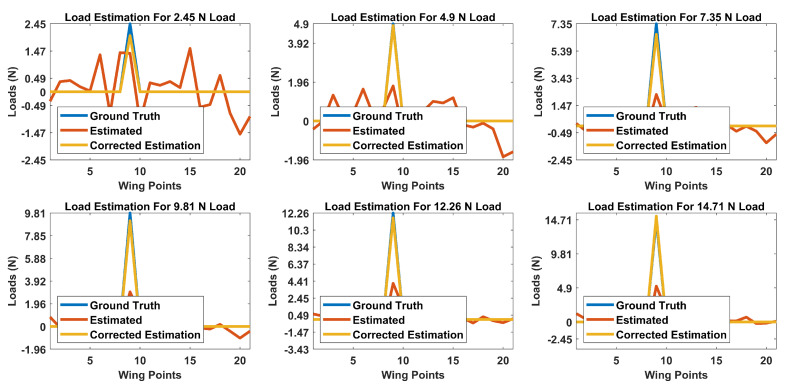
Optimized and corrected load estimations for distributed loads acting at point 9 of the wing.

## Appendix A. Localization and Estimation of Loads Based on Castigliano’s Theorem

Based on Castigliano’s second theorem [40,41], for a linearly elastic structure under loads $P_{1},P_{2},P_{3},\cdots ,P_{m}$ at points 1,2,3,⋯,n, the deflection $W_{j}$ of point *j* can be calculated as follows:

(A1) $$W_{j}=\sum_{m}a_{n\times m}P_{m}$$

where $a_{n\times m}$ is the deflection at point *n* due to a unit load applied at point *m*, normalized by the magnitude of the load. Based on this theorem, given loads at *m* points, one can calculate deflections at *n* points, as given in matrix form in Equation (A2). In order to construct *A* matrix, the concentrated loads mentioned in Section 2.1 were placed at eight different positions of the wing and the corresponding deflections *D* were recorded. Afterward, these measured deflections were normalized by the magnitude of the loads (L) acting on them to calculate the *A* matrix as given in Equation (A3). Moreover, this was performed for all load magnitudes i.e, [2.45, 4.9, 7.35, 9.81, 12.26, 14.71] N. Afterward, the average of all these magnitudes was calculated to be used as the final *A* matrix due to the nonlinear nature of the wing material as shown in Figure A1 and the inevitable sensor noise.

(A2) W=AP

(A3) $$A=\frac{D^{T}}{L}$$

where $W\in {ℜ}^{n}$, $A\in {ℜ}^{nm}$, $P\in {ℜ}^{m}$, $D\in {ℜ}^{mn}$, and L∈ℜ.

However, the objective of this work is the inverse of the above mentioned problem i.e., given deflections at *n* points, we want to calculate loads at *m* points. To do that, one can calculate the pseudo inverse of *A* to obtain *K*. The calculated *K* is shown in Table A1. This is valid since Castigliano’s first theorem is basically the inverse of its second theorem. Therefore, loads can be estimated by using the following equation:

(A4) P=KW

(A5) $$\left[\begin{array}{c} P_{1}\\ \vdots \\ P_{8} \end{array}\right]=\left[\begin{array}{c} 4244.729867\cdots -1615.667686\\ \vdots \ddots \vdots \\ -2459.317023\cdots 1233.037717 \end{array}\right]\left[\begin{array}{c} W_{1}\\ \vdots \\ W_{8} \end{array}\right]$$

where $K=A^{-1}$ is the unit load at point *m* due to the deflection measured at point *n*, normalized by the magnitude of the deflection.

**Table A1:** Calculated *K* matrix.

Deflection Measurement Points	Load Position							
	1	2	3	4	5	6	7	8
1	4244.72987	−698.685	553.1295	1970.979	−2260.34	−4685.71	−167.085	−1615.67
2	−6541.85525	1100.622	−640.224	−3025.09	3043.874	6964.995	49.58206	2560.928
3	−2694.30173	545.9633	−648.338	−1104.24	804.3221	2842.263	388.2263	979.052
4	6206.63609	−1303.48	833.578	2568.318	−2257.68	−6721.99	−652.854	−2450.1
5	3874.76045	−314.587	580.265	1808.919	−1436.91	−3625.9	344.6381	−1384.48
6	13,631.5189	−2141.29	2321.193	6042.803	−5624.8	−13,741	−375.178	−4919.03
7	−12,850.7951	3280.897	−1219.55	−5191.01	4515.127	14,360.46	1428.491	4495.81
8	−2459.31702	−152.446	−956.594	−1151.77	1369.812	2172.997	−43.5148	1233.038

After calculation of *K* matrix, one can now estimate loads at different points of the wing by providing the deflections measured at various points of the wing. Below are the results for the cases when the load is acting at point 2 of the wing with varying magnitudes of [2.45, 4.9, 7.35, 9.81, 12.26, 14.71] N, and the loads at all of the 8 points were calculated using Equations (A4) and (A5), for which the results are plotted in Figure A2.

### Appendix A.1. Load Localization Based on Castigliano’s Theorem

From the above results, it is seen that the load estimation fails as it is since it is estimating that loads with different magnitudes at different points of the wing are responsible for the measured deflections. In order to overcome this shortcoming, this work proposes the loads to be first localized, and then their magnitudes can be calculated. In order to localize the loads, the measured deflections at any time are compared with the constructed deflection *D* matrix and then the Pearson correlation [42] is used for finding the maximum correlation between them as follows:

- Given a new measurement as the one in Table A2.
  **Table A2:** New deflection measurements (mm) at 8 points of the UAV wing.
  Load Position	Deflection Measurements (mm) at 8 Points							
  	1	2	3	4	5	6	7	8
  2	3.65	4.58	5.84	7.71	2.28	4.01	5.32	7.40
- Compare the new measurements with the constructed deflection *D* matrix given in Table A3.
  **Table A3:** Constructed deflection (*D*) matrix.
  Load Position	Deflection Measurements (mm) at 8 Points							
  	1	2	3	4	5	6	7	8
  1	2.12	2.21	2.23	3.26	1.06	1.67	2.21	3.01
  2	2.54	3.09	3.67	5.19	1.30	2.50	3.70	5.19
  3	3.42	4.47	5.88	8.24	1.98	3.75	5.88	7.74
  4	4.19	5.75	7.87	10.98	2.84	5.13	8.12	10.50
  5	2.38	2.26	2.66	3.51	1.74	1.96	2.95	3.56
  6	3.13	3.03	3.91	4.79	2.32	3.07	4.49	5.14
  7	3.43	4.15	5.82	7.68	2.95	4.29	6.72	8.08
  8	4.61	5.52	7.99	10.76	3.79	5.68	8.76	11.01
- Find the maximum Pearson correlation coefficient between the current measurement given in Table A2 and the constructed deflection matrix *D* given in Table A3, which in turn will correspond to the actual load position as shown in Table A4.
  **Table A4:** Calculated Pearson correlation coefficients.
  	Load Position							
  	1	2	3	4	5	6	7	8
  **Pearson Correlation Coefficient**	0.9498	0.9920	0.9914	0.9859	0.9324	0.9354	0.9461	0.9611

From these results, it is seen that the load can be successfully localized using this method. Besides its obvious usage for load localization, this information is vital for the correct estimation of load magnitudes based on the proposed methodology for load estimation based on Castigliano’s theorem as formulated in the following subsection.

### Appendix A.2. Correction of Load Estimation Based on Castigliano’s Theorem via Localization and Optimization

Now that the loads are localized, one can correct the estimated loads by using the localization information. This can be done by first eliminating all the loads other than the localized one. Second, since the theory estimates that the measured deflections are due to a combination of loads acting at different points, one can conclude that the actual load applied at a single point is the cumulative sum of all the loads acting on the wing. Therefore, once the load is localized, its magnitude will be equal to the sum of all the estimated loads. The effectiveness of this can be seen in the results obtained in Figure A3 for loads acting again at point 2 of the wing.

However, as seen from the obtained results in Figure A3, the estimated loads’ accuracy is low in general. This is due to the calculated *K* matrix. Since the *K* matrix was calculated through pseudo inverse, it may not provide the optimal relationship between the measured deflections and the applied loads. Sensor noise also affects this, since the *A* matrix was constructed using measurements acquired at different loading cases. Therefore, this work proposes to optimize the calculated *K* matrix using a backpropagation algorithm such as Adam [20] optimizer. The initial solution to the optimizer was provided by the calculated pseudo inverse, and the optimization was defined as follows:

(A6) $$\hat{F}=\hat{K}V$$

(A7) $$CF=∥\left(F-\hat{F}\right){∥}_{2}$$

where $\hat{K}\in {ℜ}^{mn}$ is the matrix to be optimized, $V\in {ℜ}^{nN}$ is the deflection matrix with *N* samples measured by the depth sensor, $\hat{F}\in {ℜ}^{mN}$ is the estimated load matrix for *N* samples, $F\in {ℜ}^{mN}$ is the ground truth load matrix obtained from the applied loads’ magnitudes and CF is the cost function defined to be the Frobenius norm of the matrix.

The optimized $\hat{K}$ is shown in Table A5. Using this optimized matrix, the loads can now be estimated quite accurately, as shown in Figure A4. The same procedure can be applied to distributed loads by considering them as new load locations and constructing a 21×21 deflection matrix. A sample result obtained for distributed loads is shown in Figure A5, in which the deflection matrix was extended to include the new 13 distributed points.

**Table A5:** Optimized $\hat{K}$ matrix.

Deflection Measurement Points	Load Position							
	1	2	3	4	5	6	7	8
1	24.7421	−11.9939	−17.0887	30.0612	−93.1855	−65.4709	−12.1050	−17.1733
2	38.5250	−9.2518	9.0438	−6.6004	27.5545	−3.4873	−13.5474	31.7971
3	14.5861	79.1131	60.0194	23.6086	−31.8746	−5.7411	24.9256	4.4628
4	−10.9021	36.3568	−3.8216	−22.3221	24.4179	−6.5668	−4.5749	−25.8135
5	15.0917	42.9939	47.0306	35.2446	67.4271	104.4526	96.2090	18.4261
6	31.7370	−31.4353	23.4873	−21.0367	18.1937	59.4679	69.4669	21.4363
7	11.7676	135.0571	89.8542	37.6748	−9.2130	29.2120	37.8665	2.0163
8	−15.6636	−23.5698	−59.6106	−5.5923	−25.2610	70.9806	27.5923	56.5209
